# Wee1 is required to sustain ATR/Chk1 signaling upon replicative stress

**DOI:** 10.18632/oncotarget.3865

**Published:** 2015-04-19

**Authors:** Priyanka Saini, Yizhu Li, Matthias Dobbelstein

**Affiliations:** ^1^ Institute of Molecular Oncology, Göttingen Centre of Molecular Biosciences (GZMB), Faculty of Medicine, University of Göttingen, Göttingen, Germany

**Keywords:** Wee1, ATR signaling pathway, replicative stress, checkpoint kinases, gemcitabine

## Abstract

The therapeutic efficacy of nucleoside analogues, e.g. gemcitabine, against cancer cells can be augmented by inhibitors of checkpoint kinases, including Wee1, ATR, and Chk1. We have compared the chemosensitizing effect of these inhibitors in cells derived from pancreatic cancer, a tumor entity where gemcitabine is part of the first-line therapeutic regimens, and in osteosarcoma-derived cells. As expected, all three inhibitors rendered cancer cells more sensitive to gemcitabine, but Wee1 inhibition proved to be particularly efficient in this context. Investigating the reasons for this potent sensitizing effect, we found that Wee1 inhibition or knockdown not only blocked Wee1 activity, but also reduced the activation of ATR/Chk1 in gemcitabine-treated cells. Combination of several inhibitors revealed that Wee1 inhibition requires Cyclin-dependent kinases 1 and 2 (Cdk1/2) and Polo-like kinase 1 (Plk1) to reduce ATR/Chk1 activity. Through activation of Cdks and Plk1, Wee1 inhibition reduces Claspin and CtIP levels, explaining the impairment in ATR/Chk1 activity. Taken together, these results confer a consistent signaling pathway reaching from Wee1 inhibition to impaired Chk1 activity, mechanistically dissecting how Wee1 inhibitors not only dysregulate cell cycle progression, but also enhance replicative stress and chemosensitivity towards nucleoside analogues.

## INTRODUCTION

Gemcitabine (2′, 2′-difluorodeoxycytidine, dFdC), an analogue of deoxycytidine, is active against a broad spectrum of solid tumors, mostly pancreatic cancer [1], but also breast cancer [2], bladder cancer [3] or non-small cell lung cancer [4]. Pancreatic cancer is the eighth leading cause of cancer-related deaths [5]. Currently, gemcitabine is the principal compound used for its treatment, and it improves survival in a fraction of patients; however, the tumor response rate to gemcitabine monotherapy is only 5.4% [1], and the median progression-free survival under such therapy is 3.5 months [6]. Thus, in nearly all cases, pancreatic cancers display either primary or secondary resistance towards gemcitabine. This raises the need to identify strategies for improving the chemosensitivity of pancreatic cancer cells.

Cancer cells can evade the normal physiological signals controlling growth and survival by deregulating kinases. This notion initiated the design of small molecules that target and inhibit this class of enzymes [7]. Checkpoint kinases have emerged as therapeutically important targets, as their inhibition can sensitize cancer cells to DNA-damaging chemotherapeutics. In a majority of cancer cells, the G1/S checkpoint is impaired; as a consequence, these cells rely on intra S and G2/M checkpoints for DNA repair and survival [8]. Known players involved in the intra S and G2/M checkpoints include ATR, Chk1, and Wee1. Thus, combining inhibitors of these kinases with gemcitabine can sensitize tumor cells, including pancreatic, colon and breast tumors [9, 10, 11]. Gemcitabine leads to replicative stress and activates the intra S-phase checkpoint which, in turn, counteracts the damage to DNA. Therefore, inhibitors of checkpoint kinases enhance replicative stress, DNA damage, and tumor cell death. However, there is a lack of quantitative comparisons between the efficacy of inhibiting different checkpoint kinases to sensitize cells towards gemcitabine. Moreover, it remains to be determined how Wee1 and ATR/Chk1 activities affect each other in gemcitabine-treated cells.

In our study, we found that Wee1 inhibition is particularly potent to eliminate gemcitabine-treated cancer cells, as compared to the inhibition of Chk1 or ATR. Importantly, inhibition of Wee1 in gemcitabine-treated cells hampered the ATR/Chk1 pathway, thus resulting in the impairment of at least three kinases that would otherwise attenuate replicative stress. Inhibition of Cyclin-dependent kinases (Cdks) along with Wee1 rescued the ATR/Chk1 activity, thus identifying Cdks as mediators of ATR/Chk1 inactivation in this system. Furthermore, we observed that increased activity of Cdks upon inhibition of Wee1 caused activation of Polo-like kinase1 (Plk1). Plk1, in turn, led to the reduction of Claspin and CtIP levels, thereby attenuating the ATR/Chk1 pathway. These findings thus identify a cross-talk between Wee1 and ATR/Chk1 activities and a role of Wee1 in sustaining ATR/Chk1 activation during replicative stress.

## RESULTS

### Inhibitors of Chk1, Wee1 or ATR sensitize tumor-derived cells towards gemcitabine

**Figure 1:**
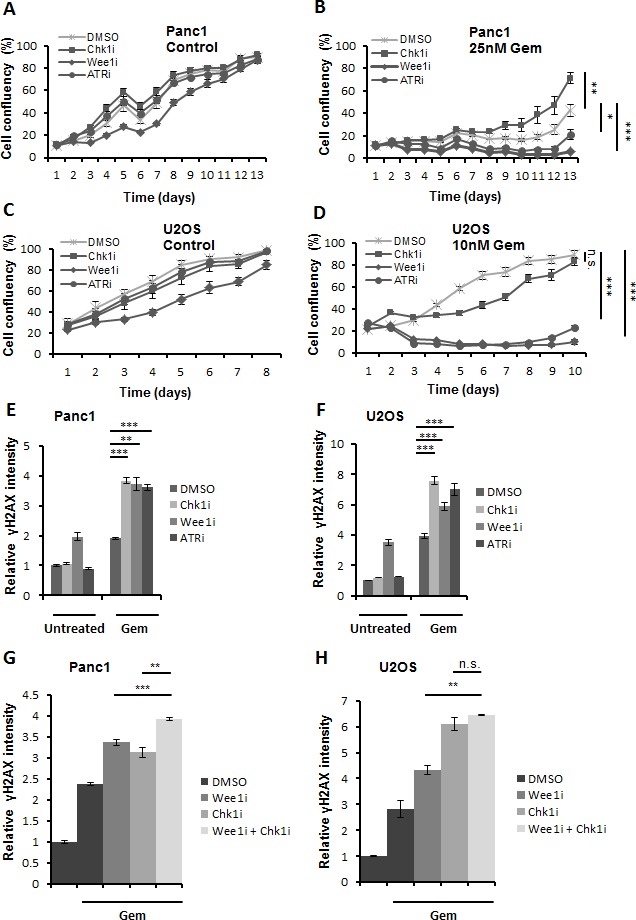
Three checkpoint kinase inhibitors cooperate with gemcitabine to enhance cytotoxicity **A.**-**D.** Panc1 and U2OS cells were treated with 2.5μM SB 218078, 0.5μM MK-1775 and 5μM VE-821 (referred to as Chk1i, Wee1i, and ATRi, respectively, for their target kinases), in the absence or presence of gemcitabine (Gem) at the indicated concentrations. After 24 h, all drugs were removed and fresh medium was added. Cells were incubated for 8-13 days, and confluency was measured each day using brightfield microscopy (Celigo cell cytometer). Error bars represent the SD, *n* = 3. *p*-values (based on Student's *t*-test, 2-sided, assuming different variances) were determined for the last measurement of respective cell line. **E, F.** Cells were treated for 24 h with gemcitabine, followed by treatment with checkpoint kinase inhibitors (5μM Chk1i; 1μM (Panc1) or 0.5μM (U2OS) Wee1i; 10μM ATRi) and gemcitabine for another 20 h. Cells were then fixed and stained for γH2AX. Detection and analysis was performed using automated immunofluorescence microscopy (BD Pathway). Error bars represent the SD, *n* = 3. Images of γH2AX stainings are shown in (Supplemental Figure S4 A, B). **G, H.** Cells were treated with 1μM Wee1i, 5μM Chk1i or DMSO in the presence of 300nM gemcitabine for 24 h. As a control, cells were treated with DMSO without gemcitabine. The cells were then processed as described in (E-F).

For comparative assessment of their chemosensitizing activities, we evaluated pharmacological inhibitors against Chk1, Wee1 and ATR (*SB 218078, MK-1775, and VE-821* respectively). The efficiency of these inhibitors was confirmed through immunoblot staining of their respective substrates (Supplemental Figure 1A, 1B). Earlier studies performed using these inhibitors have shown sensitization of tumor cells towards various chemotherapeutics [9, 11, 12, 13], here, we were aiming at the direct comparison of the cytotoxic effects of these inhibitors in combination with gemcitabine. We investigated the long-term effect of the combined treatment by monitoring the growth of the cells over 1-2 weeks after treatment. Panc1 (pancreatic adenocarcinoma) and U2OS (osteocarcinoma) cells were treated with the inhibitors in the presence or absence of gemcitabine for 24 h. After removal of all the drugs, the growth of the cells was followed using bright field microscopy and automated image analysis (Celigo cytometer) for 8-13 days. The length of the experiments was chosen as to allow control-treated cells to reach confluence. We observed that combining inhibitors of either Wee1 or ATR with gemcitabine retards the growth of the cells to a higher extent than the Chk1 inhibitor in both Panc1 and U2OS cells (Figure 1A-1D). Similarly, MiaPaCa2 (pancreatic adenocarcinoma) cells were found to be sensitized towards gemcitabine upon inhibition of Wee1 or ATR (Supplemental Figure 1C). Furthermore, cell viability assays in these cell lines revealed that combining the Wee1 inhibitor with gemcitabine leads to more pronounced cell death in comparison to single drug treatment (Supplemental Figure 1D-1F).

In parallel, we determined the phosphorylation of (the histone variant) H2AX, an established marker of DNA damage response, directly after treatment with the drugs for 24 h. We used quantitative immunofluorescence to measure the amount of phosphorylated H2AX (γH2AX). We found that the inhibition of each of the three kinases cooperates with gemcitabine in potentiating the DNA damage signal as determined by increased average γH2AX intensity (Figure 1E, 1F). To rule out that the appearance of γH2AX is a result of apoptosis [14] rather than the direct consequence of DNA damage, we performed similar experiments in the presence of Z-VAD.fmk, a pan caspase inhibitor that prevents apoptosis. However, caspase inhibition did not interfere with the accumulation of γH2AX in this context (Supplemental Figure 1G).

Wee1 inhibition increased γH2AX levels even on its own (Figure 1E, 1F) and it also proved to impair survival to a particularly large extent (Figure 1A-1D). In contrast, we observed only a mild cooperative effect on γH2AX accumulation when combining the inhibitor of Chk1 with Wee1 inhibition (Figure 1G, 1H). This observation held true even in the presence of Z-VAD.fmk (Supplemental Figure 1H). This raised the question whether the Wee1-dependent signaling pathways might be epistatic to the ATR/Chk1 pathway, or vice-versa.

### Wee1 inhibition attenuates Chk1 phosphorylation in gemcitabine-treated cells

**Figure 2:**
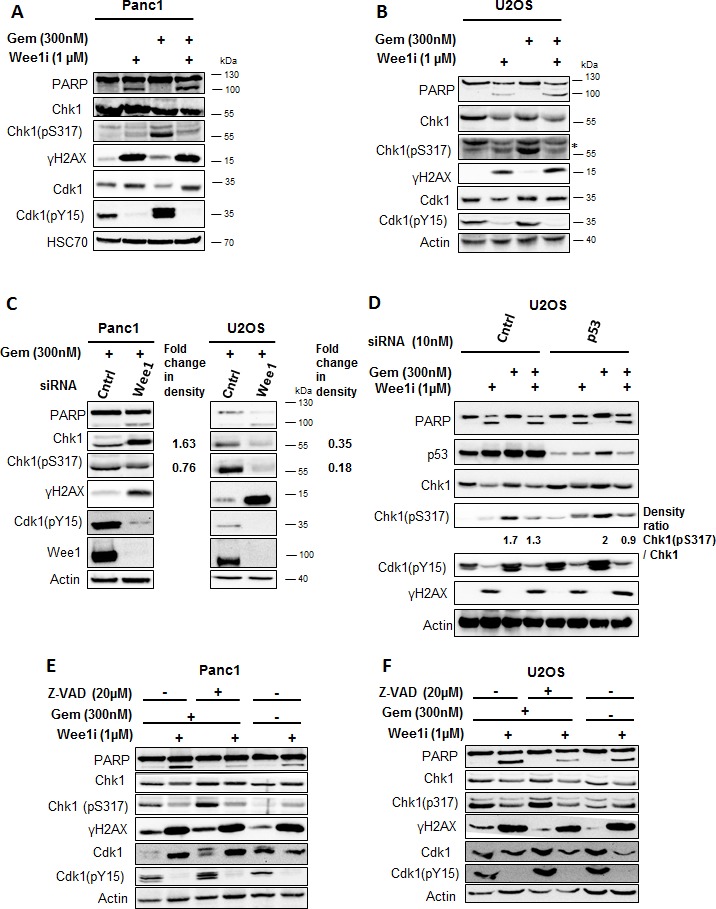
Inhibition of Wee1 decreases the phosphorylation of Chk1 in gemcitabine-treated cells **A, B.** Panc1 and U2OS cells were treated with 1μM Wee1i or DMSO, with or without 300nM gemcitabine, for 24 h. Blots of cell lysates were stained for phosphorylation of the ATR-substrate Chk1. HSC 70 or β-Actin was stained as loading controls. **C.** Cells were depleted of Wee1 by transfection with 10nM siRNA for 48h, followed by gemcitabine treatment for 24 h and immunoblot analysis as in (A, B). Scrambled siRNA was used as control. **D.** Cells were transfected with siRNA against p53 and scrambled siRNA was used as control. After 48 h (for each condition), cells were exposed to Wee1 inhibitor in the presence or absence of gemcitabine. 24 h later, cells were harvested and immunoblotting was performed. β-Actin was stained as loading control. **E, F.** Cells were treated with Wee1i or DMSO, with or without gemcitabine, in the presence or absence of the pan-caspase inhibitor Z-VAD.fmk at the indicated concentrations. After 24 h, the cells were subjected to immunoblot analysis.

To analyze the signaling pathways involved in the DNA damage response upon Wee1 inhibition, we detected DNA damage signaling intermediates through immunoblot analysis. Cells were treated with the Wee1 inhibitor and/or gemcitabine for 24 h, followed by detection of DNA damage response factors (Figure 2A, 2B). The activity of the inhibitor was verified by detecting the phosphorylation of Cdk1 at Tyr15, a known Wee1 phosphorylation site [15]. As expected, this phosphorylation was decreased upon treatment with the Wee1 inhibitor (Figure 2A, 2B). Next, we determined the activity of the ATR-Chk1 signaling pathway upon Wee1 inhibition. Phosphorylation of Chk1 at Ser317 is mediated by ATR and activates Chk1 [16]. Strikingly, we observed that Chk1 phosphorylation (Ser317) decreased upon Wee1 inhibition in gemcitabine-treated cells. To our knowledge, this is the first time that an impact of Wee1 on Chk1 activation is reported. γH2AX intensity did not decrease by Wee1 inhibition. This experiment was also performed after removing Wee1 using two distinct siRNAs, and this also reduced the phosphorylation of Chk1 in gemcitabine-treated U2OS and Panc1 cells (Figure 2C and Supplemental Figure 2A). This decreased activation of Chk1 was independent of the p53 status of the cells, since both U2OS (p53 wild type) and Panc1 (p53 mutant; R273H) [17] cells showed reduced phospho-Chk1 upon Wee1 inhibition. To further rule out a role of p53, we knocked down p53 in U2OS cells and treated them with Wee1 inhibitor, with or without gemcitabine. Eliminating p53 led to somewhat higher levels of total Chk1, in agreement with the notion that Chk1 is negatively regulated by p53 [18]. Chk1 phosphorylation was induced by gemcitabine, regardless of the p53-knockdown. Importantly, however, the phosphorylation of Chk1 was still reduced when gemcitabine-treated cells were additionally incubated with a Wee1 inhibitor, regardless of the p53 knockdown (Figure 2D). We conclude that the inhibition or removal of Wee1 hampers the Chk1 signaling pathway and leads to diminished activation of Chk1 in cells that are undergoing replicative stress.

PARP cleavage was increased when Wee1 inhibition was combined with gemcitabine, indicating caspase activity in these cells (Figure 2A-2D). To exclude that apoptosis may lead to a loss in the phosphorylation of Chk1, e.g. by general removal of phosphate groups from proteins [19] or PP2A-mediated Chk1 dephosphorylation [20, [21], we performed the treatment of the cells with gemcitabine and/or Wee1 inhibitor in the presence of Z-VAD.fmk. Analysis of the blots showed that the loss of Chk1 phosphorylation by Wee1 inhibition occurred independent of caspase activation (Figure 2E, 2F). Thus, active caspases are not required for this impairment of the ATR/Chk1 signaling axis.

### Wee1 is required for sustained ATR-Rad17 signaling in gemcitabine-treated cells

**Figure 3:**
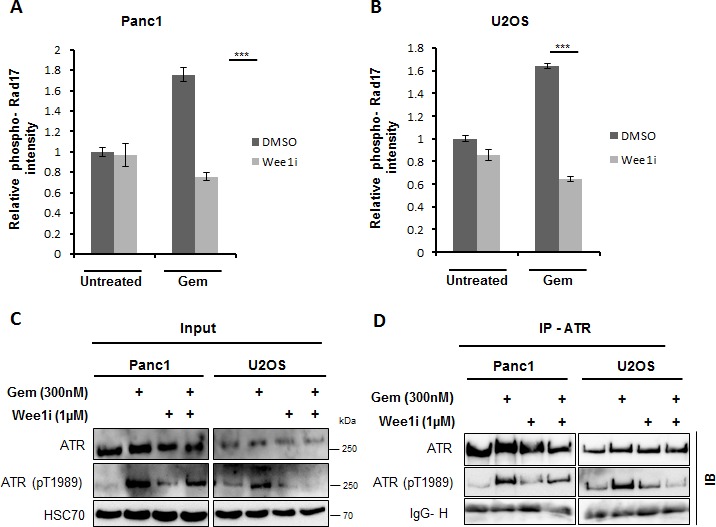
ATR activity is hampered upon inhibition of Wee1 in the presence of gemcitabine **A, B.** Panc1 and U2OS cells were treated with 1μM Wee1i or DMSO in the presence or absence of 300nM gemcitabine for 24 h. Cells were then fixed and stained for phosphorylated Rad17 (another ATR-substrate). Fluorescence intensities were determined by automated microscopy (BD Pathway). Error bars represent the SD, *n* = 3. Images of phospho-Rad17 staining are shown in (Supplemental Figure S4C, D). **C, D.** Panc1 and U2OS cells were treated with 1μM Wee1i or DMSO in the presence or absence of 300nM gemcitabine for 24 h. Cells were harvested and immunoprecipitation (IP) of ATR was performed. Phosphorylated ATR (Thr1989) was stained on immunoblots (IB), in the cell lysates (Input, C), and after ATR IP **D.** The Immunoglobulin G heavy chain (IgG-H) of the precipitating antibody was detected by the secondary IB antibody and shown as a loading control.

Besides Chk1, we also detected the phosphorylation of another ATR substrate, Rad17 (Ser645) [22] as a function of Wee1 activity. We performed quantitative immunofluorescence analysis of the phosphorylation of Rad17 upon combining the inhibition of checkpoint kinases with gemcitabine. Panc1 and U2OS cells were treated with the 1μM Wee1 inhibitor and gemcitabine for 24 h, followed by analysis of phospho-Rad17 staining intensity. The inhibition/removal of Wee1 sharply decreased phospho-Rad17 accumulation in gemcitabine-treated cells (Figure 3A, 3B and Supplemental Figure 2B, 2C).

To address whether Wee1 inhibition leads to the inactivation of ATR, we detected ATR phosphorylation at Thr-1989; phosphorylation of this site has earlier been described as a marker of ATR activity [23]. Upon treatment of cells with Wee1 inhibitor and/or gemcitabine, ATR was immunoprecipitated to concentrate this protein and then immunoblotted to detect phospho-ATR (Thr1989). Phospho-ATR levels, as expected, were increased upon gemcitabine treatment, but when gemcitabine was combined with the Wee1 inhibitor, the levels of ATR phosphorylation were reduced (Figure 3C, 3D), suggesting impaired activity of ATR. These results suggest that Wee1 activity sustains the activation of ATR pathway upon induction of DNA damage by gemcitabine.

### Wee1 inhibition impairs ATR-Chk1 signaling activity through Cyclin-dependent kinases

**Figure 4:**
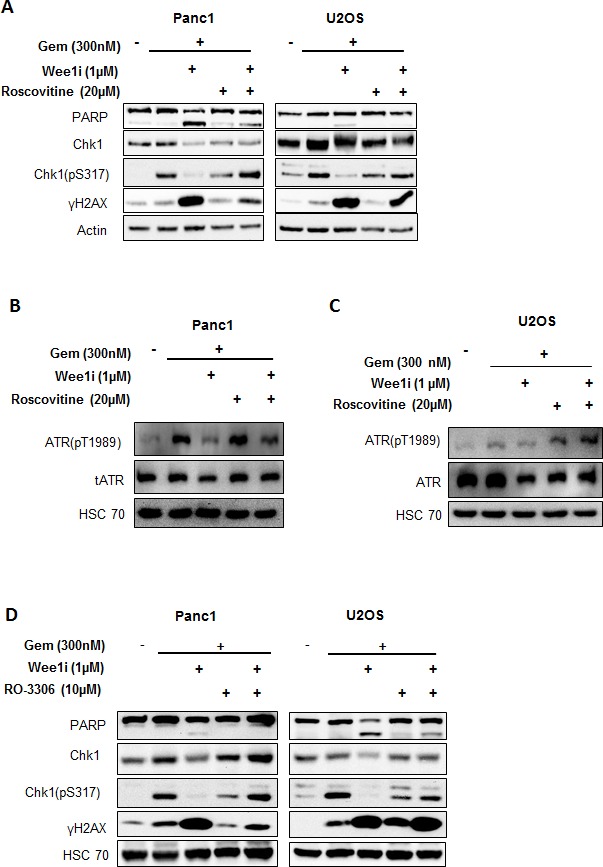
Cdks mediate the attenuation of the ATR-Chk1 pathway by Wee1 inhibition **A.** Panc1 and U2OS cells were treated with Wee1i or DMSO, with or without gemcitabine, in the presence or absence of Roscovitine (an inhibitor of Cdk1, 2 and 5) at the indicated concentrations for 24 h. Blots of the cell lysates were stained for phosphorylation of the ATR substrate Chk1. HSC 70 or β-Actin was stained as loading control. **B, C.** Panc1 and U2OS cells were treated as mentioned in (**A**). Blots of the cell lysates were stained for phosphorylation of the ATR. HSC 70 was stained as loading control. **D.** Panc1 and U2OS cells were treated with Wee1i or DMSO, with or without gemcitabine, in the presence or absence of RO-3306 (a Cdk1 inhibitor) at the indicated concentrations for 24 h. Cells were harvested and processed as in (A).

Wee1 directly phosphorylates and inhibits Cdk1 and Cdk2 at the conserved Tyr15 residue [24]. Thus, Wee1 inhibition can lead to Cdk1/2 activation. To test whether the impairment of the ATR-Chk1 pathway by Wee1 inhibition is due to Cdk activation, we inhibited Cdks using Roscovitine, along with Wee1 inhibition and gemcitabine exposure. Western blot analysis showed rescue of decreased Chk1 as well as ATR phosphorylation when Cdks were inhibited in gemcitabine-treated cells, despite the presence of Wee1 inhibitor (Figure 4A-4C). These findings imply that the inactivation of the ATR/Chk1 pathway is mediated through Cdks upon Wee1 inhibition.

Roscovitine is a potent inhibitor of Cdks and binds competitively to the ATP binding domain of these kinases [25]. To further specify the Cdk(s) involved, we used a selective inhibitor of Cdk1, RO-3306. This inhibitor has nearly 10-fold selectivity for Cdk1, as compared to Cdk2 [26]. We found that RO-3306, when combined with Wee1 inhibition and gemcitabine, could restore the phosphorylation of Chk1 (Figure 4D). In line with these observations, the removal of Cdk1 by siRNAs also restored Chk1 phosphorylation upon simultaneous knock down of Wee1 in the presence of gemcitabine (Supplemental Figure 2D, 2E). In conclusion, Cdk1 is specifically required for inactivating the ATR-Chk1 pathway upon Wee1 inhibition.

Functional inactivation of the Retinoblastoma protein (also referred as pRb) has been found to be controlled by distinct Cyclin-cdk complexes, namely Cyclin D-Cdk4/6, Cyclin E-Cdk2 and Cyclin A-Cdk2/1 [27]. As Cdks negatively regulate pRb, we tested whether pRb might be involved in maintaining the activation of the ATR signaling pathway, e.g. through E2F-mediated transcription of ATR and/or its signaling intermediates. However, the mRNA levels of ATR did not significantly change upon knockdown of Wee1 (Supplemental Figure 2F). Moreover, we analyzed the effects of Wee1 inhibition in Hela cells that contain the E7 protein from human papilloma virus 18, which can bind and inactivate pRb [28]. We treated this cell line with Cdk inhibitor, Wee1 inhibitor, and gemcitabine, alone or in combinations. We observed that even in Hela cells, Cdk inhibition could rescue the phosphorylation of Chk1 (Supplemental Figure 2G). This suggests that Wee1 inhibition interferes with ATR/Chk1 activity through Cdk1, but independently of pRb.

### Polo-like kinase 1 impedes the Chk1 activation in response to Wee1 inhibition

**Figure 5:**
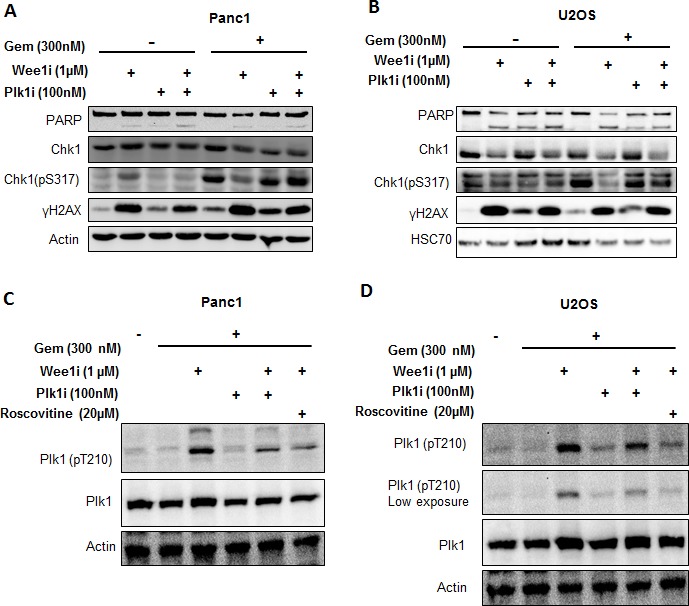
Targeting Plk1 rescues ATR-Chk1 activity in the context of Wee1 inhibition **A, B.** Panc1 and U2OS cells were treated with combinations of Wee1i, gemcitabine, and the Plk1 inhibitor, GSK 461364 (referred to as Plk1i) at 100nM for 24 h, followed by immunoblot analysis. **C, D.** Panc1 and U2OS cells were treated with Wee1i, Plk1i, and/or Roscovitine, in the presence of gemcitabine at the indicated concentrations for 8 h. Immunoblots were stained for phosphorylation of Plk1 (Thr210), an indicator of Plk1 activity.

The yeast homolog of Polo-like kinase 1 (Plk1), cdc5, is activated by the Cdk1 homolog, cdc28, in budding yeast [29, 30]. On the other hand, Plk1 is known to down-regulate the ATR/Chk1 pathway at different levels. Plk1 phosphorylates Claspin and marks it for degradation by SCFbetaTrCP, thereby restraining Chk1 activation [31, 32]. Furthermore, Plk1 interferes with CtIP activity [33]. To investigate the role of Plk1 in the negative regulation of ATR/Chk1 activity, we incubated cells with a Plk1 inhibitor (*GSK 461364*) or siRNA against Plk1, in the presence of the Wee1 inhibitor and gemcitabine; through immunoblot analysis, it was found that the inhibition or removal of Plk1 could recover the phosphorylation of Chk1 (Figure 5A, 5B and Supplemental Figure 2H, 2I). Hence, Plk1 activity is required for the attenuation of ATR/Chk1 signaling upon Wee1 inhibition.

To validate the activation of Plk1 upon Wee1 inhibition, and its dependence on Cdks, we performed western blot analysis to detect the phosphorylation of Plk1 at Thr210, a hallmark of Plk1 activation [34]. Phosphorylated Plk1 (Thr210) increased with Wee1 inhibition, but this phosphorylation vanished when inhibitors of Plk1 or Cdks were added (Figure 5C, 5D). We conclude that Plk1 activity is increased upon Wee1 inhibition in the presence of gemcitabine, and that this activation is a necessity for impeding the ATR/Chk1 pathway.

### Upon Wee1 inhibition, Plk1 mediates inactivation of Chk1 through reduction in the levels of Claspin protein

**Figure 6:**
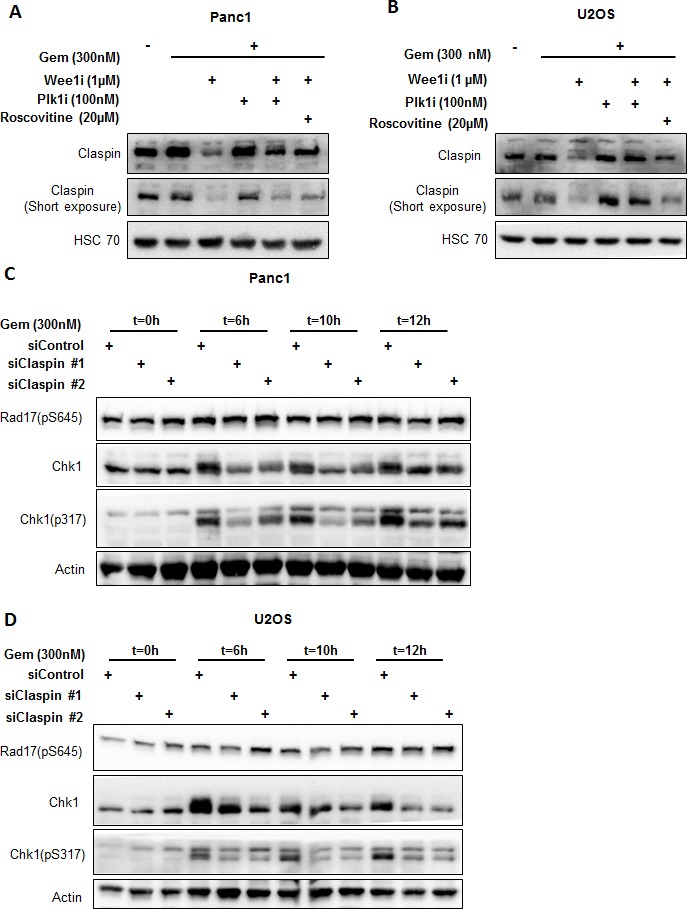
Reduced Claspin levels interfere with Chk1 activation **A, B.** Panc1 and U2OS cells were treated with Wee1i, Plk1i and/or Roscovitine, in the presence of gemcitabine, for 8 h. Blots were stained for total levels of Claspin. HSC 70 was stained as loading control. **C, D.** Claspin was knocked down by transfecting the cells with 10nM siRNAs for 48 h, followed by treatment with 300nM gemcitabine. The cells were harvested at 0 h, 6 h, 10 h and 12 h after gemcitabine addition. Immunoblots were stained for Chk1 and Rad17 phosphorylation. β-Actin was stained as a loading control.

Next, we investigated whether Wee1 inhibition diminishes Chk1 activity by altering levels of Claspin, a cofactor of Chk1 activation. We determined the levels of Claspin while inhibiting Wee1 as well as Plk1 or Cdks. Indeed, Claspin levels were decreased upon Wee1 inhibition in the presence of gemcitabine, but the original amount of Claspin was restored when inhibitors of Plk1 or Cdks were added (Figure 6A, 6B). We further observed that the decrease in the protein levels of Claspin was due to proteasomal degradation, since exposure to MG132 (a proteasome inhibitor) could reinstate the normal amount of this protein (Supplemental Figure 3A). At the same time, mRNA levels of Claspin did not change significantly upon Wee1 inhibition (Supplemental Figure 3B, 3C). Moreover, the siRNA-mediated removal of Claspin reduced Chk1 phosphorylation as well (Figure 6C, 6D). The knockdown efficiency of siRNAs was determined using immunoblot analysis (Supplemental Figure 3D). Thus, the reduction of Claspin occurs through Cdks and Plk1, and it contributes to the attenuation of Chk1 activity upon Wee1 inhibition.

### Wee1 inhibition diminishes levels of CtIP in a Cdk-dependent manner, and this hampers ATR activation upon replicative stress

**Figure 7:**
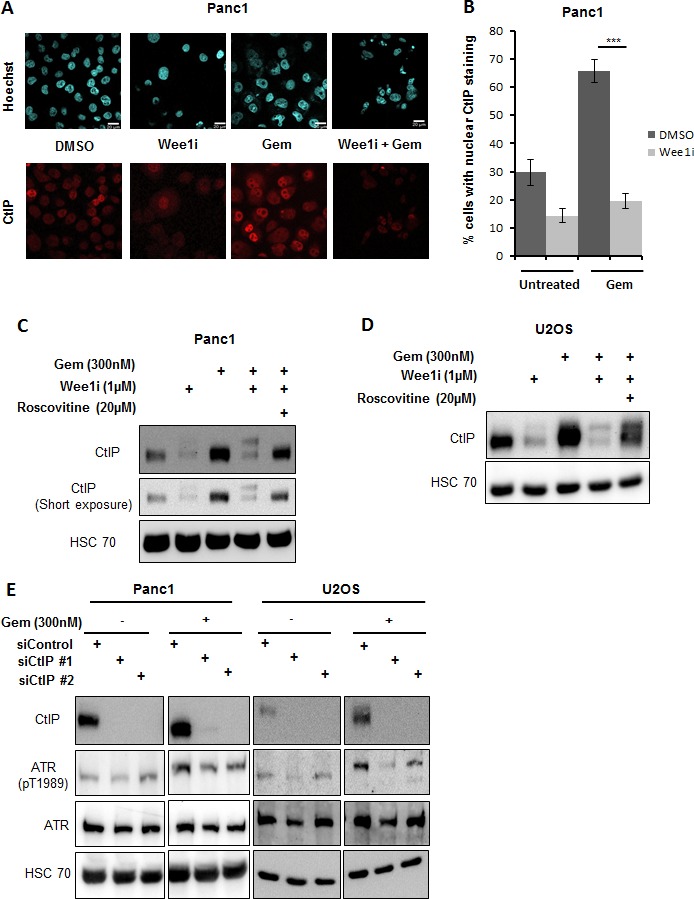
Reduction in CtIP protein levels attenuates ATR activation **A, B.** Panc1 cells were treated with combinations of Wee1 inhibitor and gemcitabine for 24 h. The cells were fixed and stained for CtIP by immunofluorescence. Images were taken using confocal microscopy **A.** Quantitative analysis was done by evaluating at least 100 cells per sample **B.** Error bars represent the SD. Scale bar represents 20μm. **C, D.** Panc1 and U2OS cells were treated with combinations of Wee1 inhibitor, Roscovitine and gemcitabine for 24 h. Blots were stained for CtIP. HSC 70 was stained as a loading control. **E.** Panc1 and U2OS cells were transfected with two different siRNAs against CtIP and negative control siRNA. After 48 h, cells were treated with 300nM gemcitabine and harvested at 24 h after gemcitabine addition. Immunoblots were stained for ATR phosphorylation. HSC 70 was used as a loading control. In the figure, immunoblots with and without gemcitabine for each cell line belong to the same blot.

The CtIP protein can act as a cofactor in ATR activation [35]. On the other hand, at least in budding yeast, CtIP has been found regulated by Plk1 [33], suggesting that Plk1 may govern ATR activity through CtIP. To test this, we determined the levels of CtIP upon Wee1 inhibition in the presence of gemcitabine. Indeed, CtIP levels decreased when Wee1 was inhibited in gemcitabine-treated cells. This was found by immunofluorescence (Figure 7A, 7B) as well as immunoblot analysis (Figure 7C, 7D). The decrease in CtIP levels could be rescued by simultaneous inhibition of Cdks through Roscovitine (Figure 7C, 7D), suggesting a role of Cdks in the reduction of CtIP levels. We further tested if removal of CtIP was sufficient to attenuate ATR activation in this context. Knockdown of CtIP using siRNAs in the presence of gemcitabine decreased ATR activation (Figure 7E). Thus, CtIP is indeed required to maintain the activity of ATR. In conclusion, the decrease in CtIP in response to Wee1 inactivation contributes to the impairment of ATR activity.

### Plk1 activation and Claspin/CtIP reduction precede the inactivation of ATR/Chk1 upon Wee1 inhibition

**Figure 8:**
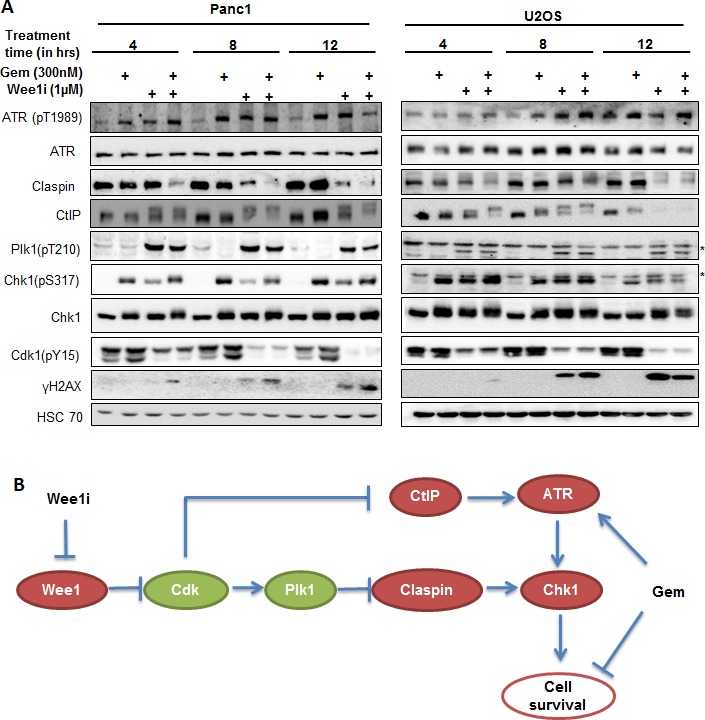
Kinetics of ATR/Chk1 attenuation upon Wee1 inhibition **A.** Panc1 and U2OS cells were treated with 1μM Wee1i or DMSO, with or without 300nM gemcitabine, for 4, 8 or 12 h. Blots of cell lysates were stained for phospho-ATR, Claspin, CtIP, phospho- Plk1, phospho-Chk1 and γH2AX, HSC 70 or β-Actin was stained as loading controls. **B.** Schematic representation of the mechanisms by that Wee1 inhibition impairs ATR-Chk1 signaling, as suggested by the results of our study. Inhibition of Wee1 activates Cdks, which in turn increases the activity of Plk1. Plk1 destabilizes Claspin and thereby impairs Chk1 activity. Cdks also mediate a reduction in CtIP levels, thus attenuating ATR activation and further contributing to the loss in Chk1 activation.

In order to understand the chronological order of the events regulating ATR/Chk1 activity, we treated Panc1 and U2OS cells with the Wee1 inhibitor and/or gemcitabine and harvested at different time points for immunoblot analysis. We observed that inhibition of Wee1 in gemcitabine-treated cells initially activated Plk1, reduced Claspin levels and altered the electrophoretic mobility of CtIP (compatible with a posttranslational modification). At a later time, the phosphorylations of ATR and Chk1 were reduced (Figure 8A; cf. Figure 2A and 2B, for 24 h treatment results). This sequence of phosphorylation events is compatible with a model depicted in Figure 8B, reaching from Wee1 inhibition through Cdk and Plk1 activation to a reduction in the activating phosphorylations of ATR and Chk1.

## DISCUSSION

The Wee1 inhibitor, MK-1775, sensitizes tumor cells towards gemcitabine with particular efficiency, even when compared to inhibitors of ATR and Chk1. MK-1775 increased H2AX phosphorylation and markedly reduced long-term survival of the cells. Mechanistic analyses then revealed that Wee1 signaling is epistatic in relation to ATR/Chk1 activity in gemcitabine-treated cells.

Thus, we observed attenuation of the ATR/Chk1 pathway upon Wee1 inhibition. This provides an attractive explanation for the observed increase in the DNA damage response when combining gemcitabine with a Wee1 inhibitor. ATR and Chk1 activity, at least in general, attenuate replicative stress [36]. Therefore, if Wee1 inhibition impairs ATR/Chk1 activity, the expected consequence is that replicative stress is enhanced, especially in the presence of a false-incorporated nucleoside analogue. In the absence of sufficient ATR/Chk1 activity, DNA replication forks tend to stall and eventually collapse [34, 37]. In such a scenario, the intermediates of incomplete DNA replication trigger a DNA damage response, e.g. through activation of ATM and/or DNA-PK. As a result, phosphorylated H2AX accumulates and cell survival is impaired. We therefore propose that attenuated ATR/Chk1 represents at least one of the reasons why Wee1 inhibitors can synergize with a number of chemotherapeutics to trigger cancer cell death [38].

Regulation of Wee1 by Chk1 has been studied, revealing that Chk1 phosphorylates Wee1 to inhibit Cdc2 phosphorylation at Tyr15 [39]. Vice versa, however, it has hitherto not been known whether and how Wee1 supports ATR signaling. Since Wee1 sustains Chk1 activity upon replicative stress (our study), whereas Chk1 diminishes Wee1 activity [39], it is tempting to speculate that a negative feedback loop limits the activation of Chk1 by Wee1.

Our study revealed that Cdks are required for negatively regulating the ATR/Chk1 pathway upon Wee1 inhibition. This is conceivable since Wee1, when active, mediates an inhibitory phosphorylation on Cdk1/2. But how would enhanced Cdk activity attenuate ATR/Chk1 signaling? Our results show that this is mediated through Plk1. In human cells, Cdk1 has been reported to ‘prime’ the Plk1 substrates by phosphorylating them. The ‘primed’ substrates, e.g. Vimentin, are then recognized and phosphorylated by Plk1 [40]. In *S. cerevisiae,* Cdk1 has been proposed to maintain the stability of Plk1 by phosphorylation at Thr23 [30], but it is currently unknown whether such a mechanism exists in the human system as well. However, we observed that phosphorylation of Plk1 at Thr210, a marker for its activation [34], increases upon Wee1 inhibition in gemcitabine-treated cells. It thus appears conceivable that Cdk activity may support Plks by more than one mechanism, enhancing its general activity as well as priming specific substrates. Once activated, we propose that Plk1 attenuates ATR/Chk1 signaling. Plk1 has been implicated in the phosphorylation and subsequent degradation of Claspin, thereby preventing the activation of Chk1 in response to replicative stress [31, 39, 32, 40].

The removal of Claspin provides an explanation for attenuated Chk1, but not for diminished ATR activity upon Wee1 inhibition. In recent studies, Wee1 inhibition has been demonstrated to impair homologous recombination [43, 44] and CtIP plays a key role in this mode of DNA repair [45]. In agreement with these findings, we observed that CtIP was degraded when Wee1 was inhibited in gemcitabine-treated cells. CtIP is phosphorylated by Cdks [46] and, at least in yeast, also by Plk1 [33]. This phosphorylation mediates binding of the peptidyl-prolyl isomerase Pin1 to CtIP. Pin1-catalyzed isomerization of CtIP facilitates the degradation of the latter [47]. Moreover, Plk1 phosphorylates and stabilizes Pin1 [47]. Therefore, we propose that upon inhibition of Wee1, hyperactive Cdks phosphorylate CtIP, while activated Plk1 stabilizes Pin1, which together facilitates proteasomal degradation of CtIP. On the other hand, CtIP is required for sustained ATR/Chk1 signaling and for keeping up the intra-S phase checkpoint [48]. As a net result, Wee1 inhibition attenuates the activities of ATR and Chk1. Taken together, our analyses reveal a pathway that leads from Wee1 inhibition to the impairment of ATR and Chk1 in the context of replicative stress (Figure 8B). Despite the attenuation of Chk1 by Wee1 inhibition, a number of studies still found cooperative effects when using inhibitors of Chk1 and Wee1 simultaneously for cancer treatment [22, 45]. We propose that the reason for this cooperativity might consist in the timing of the enzymatic activities. For optimum sensitization, it may be advantageous to block Chk1 immediately when cells are exposed to nucleoside analogues. In any case, however, our observations suggest that Chk1 and ATR are eventually attenuated by Wee1-inhibitors alone in the context of gemcitabine treatment.

Wee1 inhibitors represent promising anti-cancer drug candidates [11, 46] and are currently being tested in clinical trials of phases I and II (NCI Clinical Trials). Our results strongly suggest that Wee1 inhibition eliminates cancer cells not only by premature activation of chromosome separation [13] but also by enhancing replicative stress through impairment of ATR/Chk1 signaling. This unique combination of cytotoxic mechanisms, triggered through a single target, provides an attractive explanation for the remarkable cytotoxic efficacy of Wee1 inhibitors.

## MATERIALS AND METHODS

### Culturing of human cancer cell lines

Panc1 (human pancreatic epithelioid carcinoma) and U2OS (human osteosarcoma) cells were cultured in DMEM (Gibco, Life Technologies) with 10% FCS (Gibco, Life Technologies), 200μM L-glutamine (Gibco, Life Technologies) and antibiotics – 50U/ml Penicillin and Streptomycin (Gibco, Life Technologies), 20μg/ml Tetracycline (Gibco, Life Technologies) and 10μg/ml Ciprofloxacin (Bayer).

### Transfection of cells with siRNA, and inhibitor treatment

To knock down genes of interest, reverse transfection was performed in 6-well plates with 10nM siRNA and Lipofectamine 2000 (Life Technologies). Cells were either harvested or treated with chemicals after 48 h. siRNAs to Wee1 (s21, silencer select), Claspin #1 (s34330, silencer select), Claspin #2 (s34331, silencer select), CtIP #1 (s11849, silencer select), CtIP #2 (s11851, silencer select), p53 (s605, silencer select), Wee1 (404, silencer), Cdk1 #1 (s464, silencer select), Cdk1 #2 (s465, silencer select), Plk1 (s449, silencer select) and Negative Control No.1 siRNA (silencer select, silencer) were obtained from Ambion, Life Technologies. The following chemical inhibitors were used: Wee1 inhibitor MK-1775 (Selleckchem), ATR inhibitor VE-821 (Selleckchem), Chk1 inhibitor SB 218078 (Calbiochem, Merck), Cdk1, 2 and 5 inhibitor Roscovitine (Cell Signaling), Cdk1 inhibitor RO-3306 (Sigma Aldrich).

### Cell proliferation assay

To track cell proliferation, the *Celigo cell cytometer* (Cyntellect, San Diego, CA, United States) was used; the confluency of the cells was measured by transmission microscopy. Cells were seeded in 96- well plates (5000 cells per well). After 24 h, the confluency of the cells was measured (labeled as Day 0), followed by treatment with 0.5μM MK-1775 / 2.5μM SB 218078 / 5μM VE-821 without or with gemcitabine at the indicated concentrations. After 24 h, all the drugs were removed. Subsequent measurements of cell confluency were made after every 24 h, and media was changed every 48 h.

### CellTiter-Glo® luminescent cell viability assay

This assay (Promega) was performed to determine the amount of metabolically active cells present in a culture. It is based on the activity of luciferase, which uses ATP from cells to generate a luminescent signal, quantified by a *DLReady™Centro LB 960* luminometer. Cells were seeded in opaque-walled 96-well plates (3000 cells per well) and exposed to drugs after 24h. 72 hours later, cell lysates were prepared and luminescence was recorded.

### Preparation of whole cell lysates for SDS-PAGE

Cells were seeded in 6-well plates (1.6 × 10^5^ cells per well) for the drug treatment. Cell lysates were prepared on ice. The cells were scraped off into the medium and pelleted by centrifugation at 1500xg for 3 min at 4°C, followed by one wash in PBS. The cells were resuspended in 100μl RIPA lysis buffer (1% Triton X, 1% Desoxycholate, 0.1% SDS, 150 mM NaCl, 10 mM EDTA, 20 mM Tris-HCl pH 7.5, 100.000KIE Aprotinin) freshly supplemented with 2M urea, 1mg/ml leupeptine/aprotinine, 0.1M pepstatin A, 0.1M pefabloc. After 20 min of shaking at 4°C, the lysates were centrifuged at 15,700xg for 10min. Bicinchoninic acid (BCA) assay was used to normalize the concentration of proteins in the supernatant. The samples were then boiled with Laemmli buffer, followed by SDS-PAGE.

### Western blot analysis

Blots on nitrocellulose or PVDF membranes were stained with the following antibodies. phosphorylated Ser 139 H2AX (05-636, Millipore), phosphorylated Ser 317 Chk1 (2344, Cell Signaling Technology), phosphorylated Ser 645 Rad17 (6981, Cell Signaling Technology), phosphorylated Tyr 15 Cdk1 (ab47594, abcam), phosphorylated Thr 1989 ATR (EVU001, Kerafast), PARP (9542, Cell Signaling Technology), total Rad17 (sc-17761, Santa Cruz Biotechnology), total Chk1 (2360, Cell Signaling Technology), total Cdk1 (9116, Cell Signaling Technology), total ATR (sc-1887, Santa Cruz Biotechnology), HSC 70 (sc-7298, Santa Cruz Biotechnology), Wee1 (4936, Cell Signaling Technology), beta-Actin (ab6276-100, abcam), Claspin (2800, Cell Signaling Technology), phosphorylated Thr 210 Plk1 (558400, BD Pharmigen), total Plk1 (37-7000, Life Technologies), CtIP (61142, Active Motif), phospho-H3 (3377, Cell signaling). Secondary antibodies coupled to horseradish peroxidase (Jackson Immunoresearch) were used for chemiluminescent detection (Millipore).

### Immunoprecipitation

Cells were seeded in a 10 cm petri dish (8 × 10^5^ cells per dish). 24 h after seeding, the cells were treated with the indicated inhibitors in the presence or absence of gemcitabine for 24 h. Protease inhibitors (complete (mini) inhibitor mix from Roche) and phosphatase inhibitors (10mM NaF, 2mM Na-pyrophosphate, 1mM Na-orthovanadate) were added to the IP-lysis buffer (50mM Tris- HCl, pH 7.5, 300mM NaCl, 1% NP-40, 0.1% Na-deoxycholate) just before its use. Cells in IP-lysis buffer were scraped off the plate and transferred to an Eppendorf tube, followed by homogenization with a 26G syringe, sonication and centrifugation. 2 μg of antibody was added and incubated overnight at 4°C on a rotor. 30 μl of equilibrated Protein G sepharose beads were put in the lysates and incubated 1 h at 4°C. After 5 washes in 800 μl IP-lysis buffer, 30μl of 6 X Laemmli buffer was added to the pellet and boiled at 95°C for 5 min. The samples were subjected to SDS-PAGE and immunoblot analysis.

Immunoprecipitation was performed to concentrate ATR using the anti-ATR (N-19) antibody from Santa Cruz and then immunoblotted to determine the levels of phospho-ATR (T1989) (Kerafast).

### Immunofluorescence analysis

For immunofluorescence microscopy, the automated microscope *Pathway 855* (Becton Dickinson, Franklin Lakes, NJ, United States) was used to read fluorescence intensity in 96-well plates. For confocal microscopy, *LSM 510* laser scanning microscope (Carl Zeiss, Germany) was used.

The cells were fixed in 3.7% paraformaldehyde for 20 min, followed by permeabilization with 0.5% triton-X in PBS for 15 min and blocking for 15 min using blocking solution (3% BSA in PBS). The primary antibody to phospho-H2AX (05-636, Millipore)/phospho-Rad17 (6981, Cell Signaling Technology)/CtIP (61142, Active Motif), diluted in blocking solution, was added for 1 h, followed by incubation with a secondary antibody (Alexa-Fluor 546/488) and Hoechst 33342 (Invitrogen) diluted in blocking solution for 45 min.

Images were captured and analyzed using the BD Pathway software, wherein the region of interest (ROI), in this case the cell nuclei, were defined by Hoechst stain, and the average intensity of the antibody-coupled fluorescence within each ROI was determined.

### Cell cycle analysis by flow cytometry

Cells were seeded in 6-well plates and treated with the Wee1 inhibitor in the presence or absence of gemcitabine. After fixation in ethanol, the cells were washed in wash solution (0.05% Triton-X in PBS), followed by incubation in staining solution (2% FCS, 0.2% Triton-X in PBS) with phospho-H3 antibody (3377, Cell signaling) for 2 h and then with secondary antibody (Alexa-Fluor 488) for one hour. Subsequently, the cells were resuspended in 0.5 mg/ml RNAse A solution andincubated for 30 min at 37°C. Directly before measurement, propidium iodide (final concentration: 30 μg/ml) was added. Samples were measured using a *FACS machine Guava PCA-96 Base System* (*Millipore, Merck, Darmstadt, Germany*).

### Reverse transcription and real time quantitative polymerase chain reaction (qRT-PCR)

Total RNA from human cells was isolated. Reverse transcription was performed using M-MuLV Reverse transcriptase (New England Biolabs) and a mixture of anchored dT primers (dT_23_VN) and random nonamers. Quantitative PCR was carried out using thermostable Taq DNA polymerase (Fermentas, Thermo Scientific) in the presence of Sybr green. Fluorescence intensities were measured to determine the Ct values. The relative concentrations of mRNAs were calculated by the 2^−ΔΔCt^ method, using GAPDH or 36B4 mRNAs as references.

### Statistical analysis

Statistical significance was determined using the unpaired, two-tailed Student's T-test. Significance was assumed for *p*-values below 0.05. Asterisks in figures indicate resulting p-values as follows: *p < 0.05, **p < 0.01, ***p < 0.001. *n.s.* = not significant. *n* in figure legends indicates the number of independent experiments.

## SUPPLEMENTARY MATERIALS, FIGURES


