# Supplementary material for: Wee1 is required to sustain ATR/Chk1 signaling upon replicative stress
Source: Oncotarget. 2015 Apr 19;6(15):13072–87. doi: 10.18632/oncotarget.3865 (PMC4537000; doi:10.18632/oncotarget.3865)
Supplement: Supplementary file 1 [file oncotarget-06-13072-s001.pdf]

Wee1 is required to sustain ATR/Chk1 signaling upon replicative stress

Supplementary Material

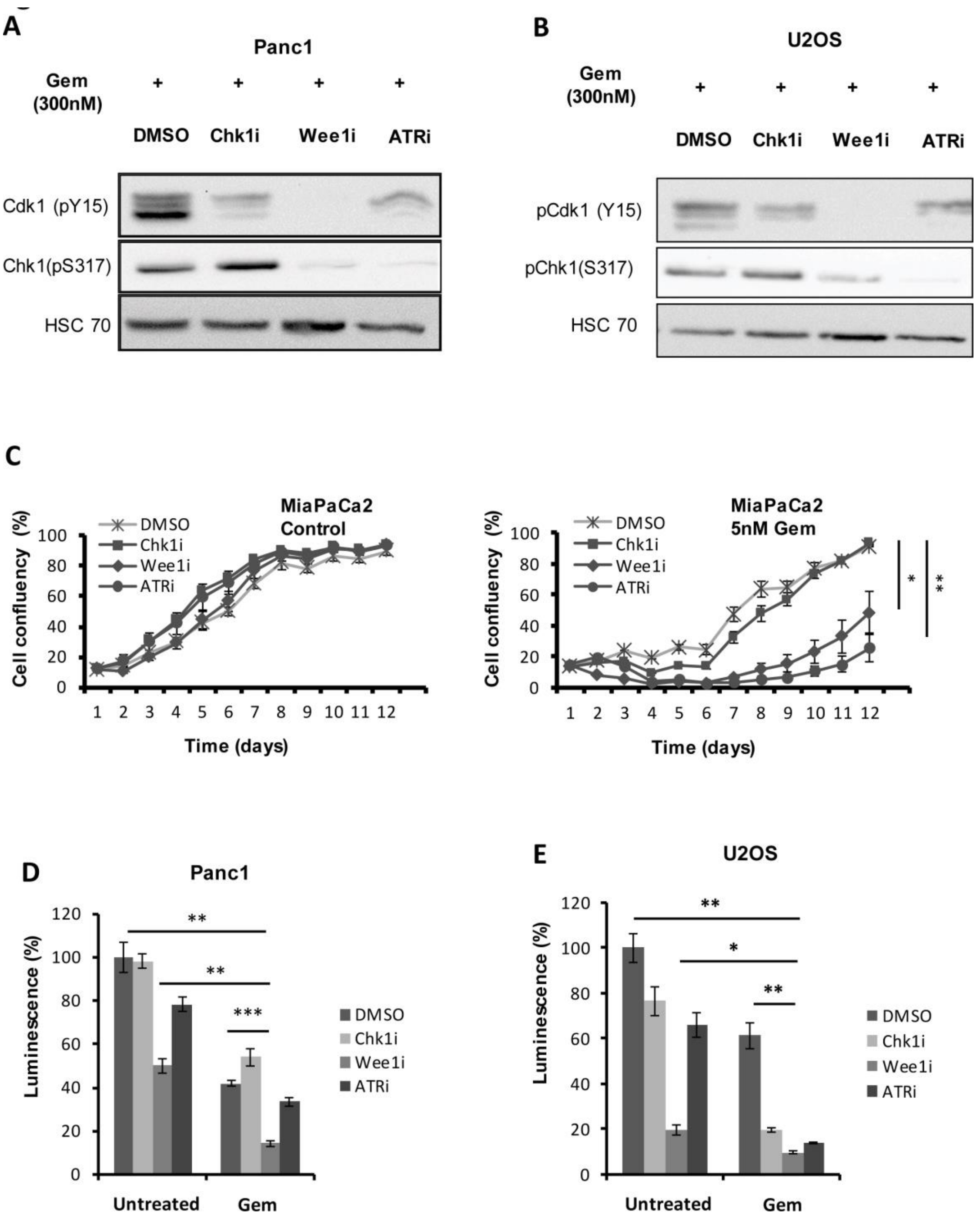

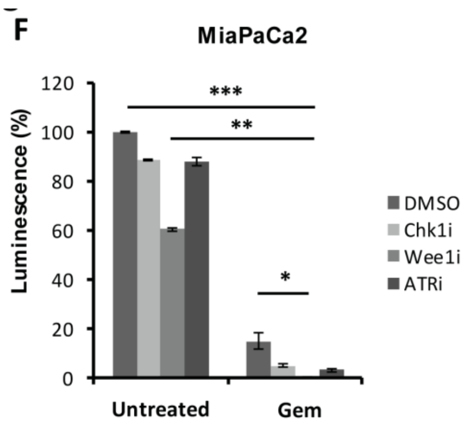

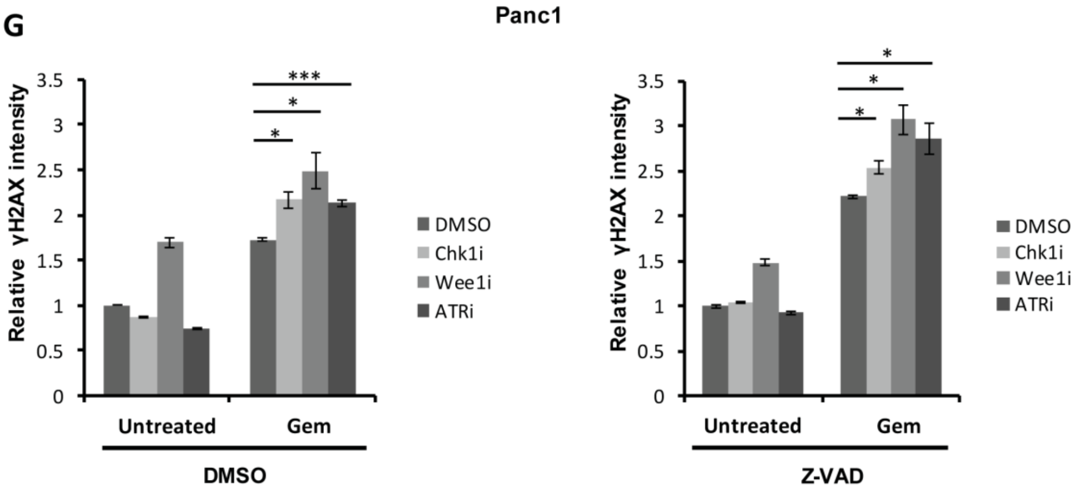

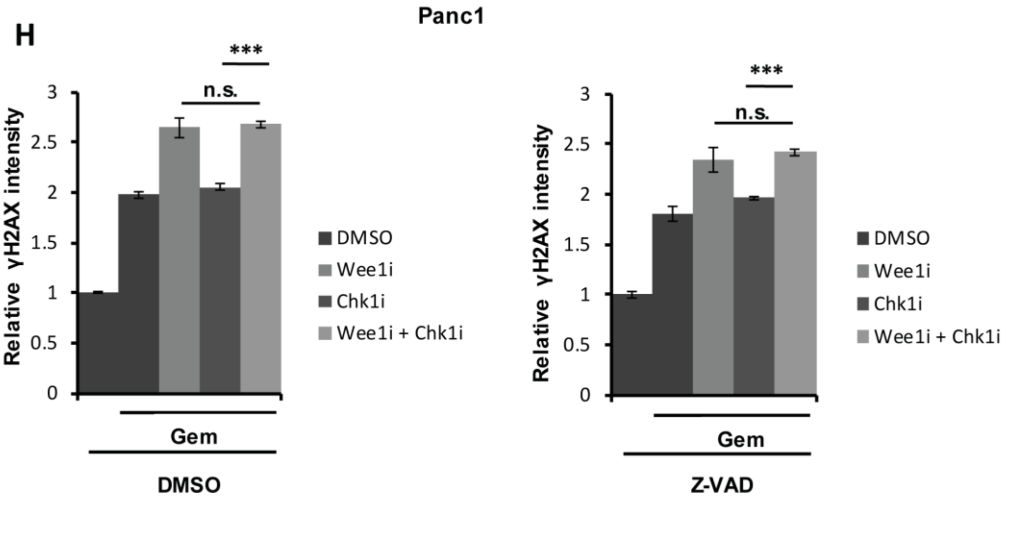

Figure S1.

(A, B) The checkpoint kinase inhibitors efficiently inhibit their target kinases.

Panc1 and U2OS cells were treated with 5µM Chk1i, 1µM Wee1i and 10µM ATRi in the presence of 300nM gemcitabine for 24 h. Blots were stained for the phosphorylation of substrates of each kinase- Cdk1 for Wee1 and Chk1 for ATR. HSC 70 was stained as loading control. Chk1 controls Cdk activity through phosphorylation of Cdc25 [51].

(C) Wee1/ATR inhibition in the presence of gemcitabine hinders cell proliferation in MiaPaCa2 cells. MiaPaCa2 cells were treated as in **Fig.1 (A-D)**. 24h later, the drugs were removed and fresh medium was added. Subsequently, cell confluence was monitored by transmission microscopy using a Celigo cell cytometer, for 12 consecutive days. Error bars represent the SD, n=3.

(D-F) Combination of Wee1 inhibitor with gemcitabine cooperates to reduce the viability of Panc1, U2OS and MiaPaCa2 cells. Cells were treated with 2.5µM Chk1i, 0.5µM Wee1i and 5µM ATRi with or without gemcitabine for 72h (Panc1, MiaPaCa2 – 5nM gemcitabine, and U2OS – 1nM gemcitabine). The cells were lysed in CellTiter-Glo® reagent, followed by measurement of the ATP content through luminescence intensity. Error bars represent the SD, n=3.

(G, H) Caspase cleavage is not responsible for increased γH2AX accumulation upon combination of Chk1/Wee1/ATR inhibition with gemcitabine. Cells were treated as outlined in **Fig.1E** and/or **Fig. 1G** with addition of either DMSO or 20µM of the caspase inhibitor Z-VAD. γH2AX was quantified by immunofluorescence analysis. n=3.

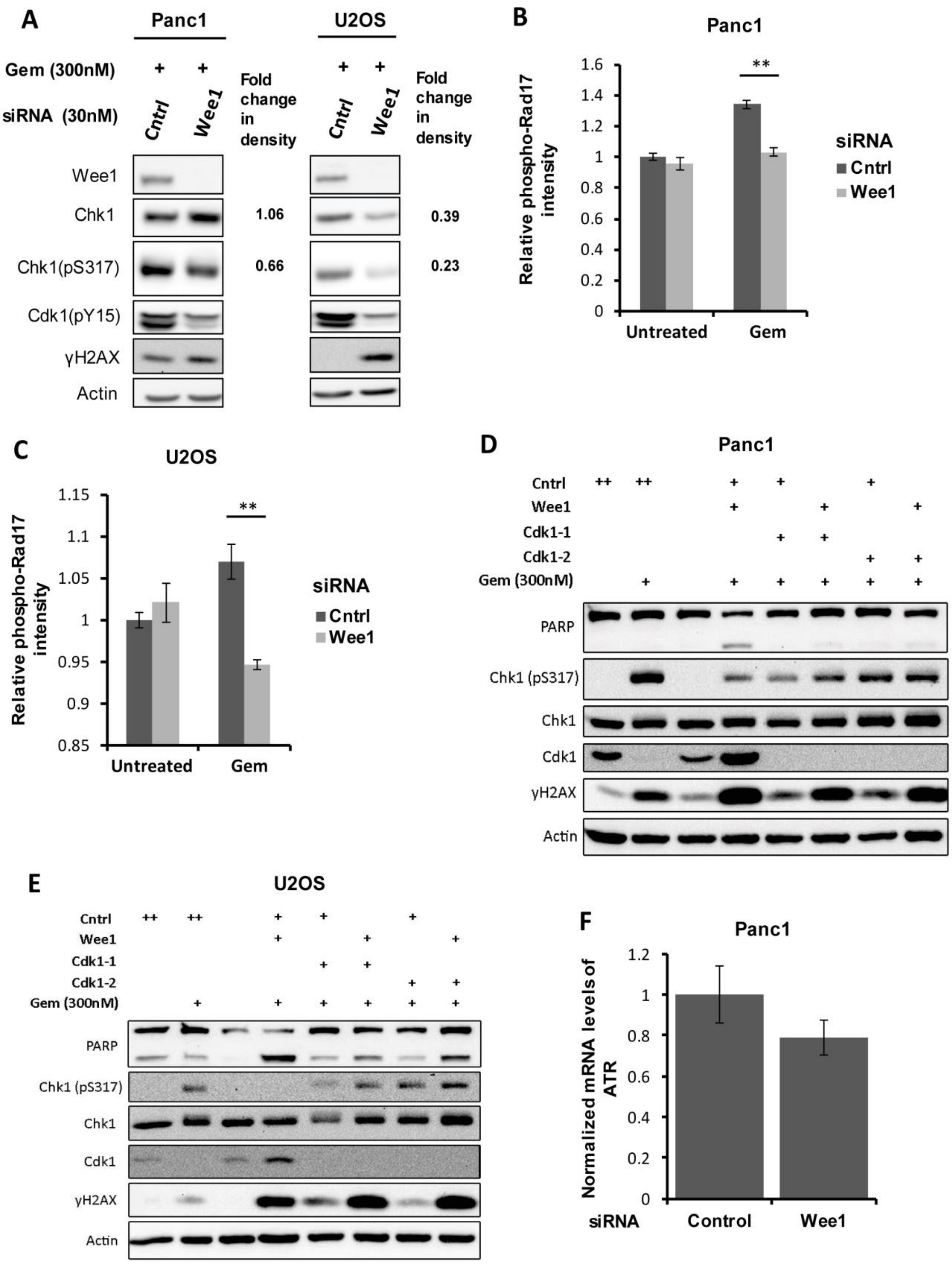

A
Panc1
U2OS
Gem (300nM)
siRNA (30nM)
Cntrl
Wee1
Fold change in density
Wee1
Chk1
1.06
0.39
Chk1(pS317)
0.66
0.23
Cdk1(pY15)
γH2AX
Actin
B
Panc1
Relative phospho-Rad17 intensity
**
siRNA
Cntrl
Wee1
Untreated
Gem
C
U2OS
Relative phospho-Rad17 intensity
**
siRNA
Cntrl
Wee1
Untreated
Gem
D
Panc1
Cntrl
Wee1
Cdk1-1
Cdk1-2
Gem (300nM)
PARP
Chk1 (pS317)
Chk1
Cdk1
γH2AX
Actin
E
U2OS
Cntrl
Wee1
Cdk1-1
Cdk1-2
Gem (300nM)
PARP
Chk1 (pS317)
Chk1
Cdk1
γH2AX
Actin
F
Panc1
Normalized mRNA levels of ATR
siRNA
Control
Wee1

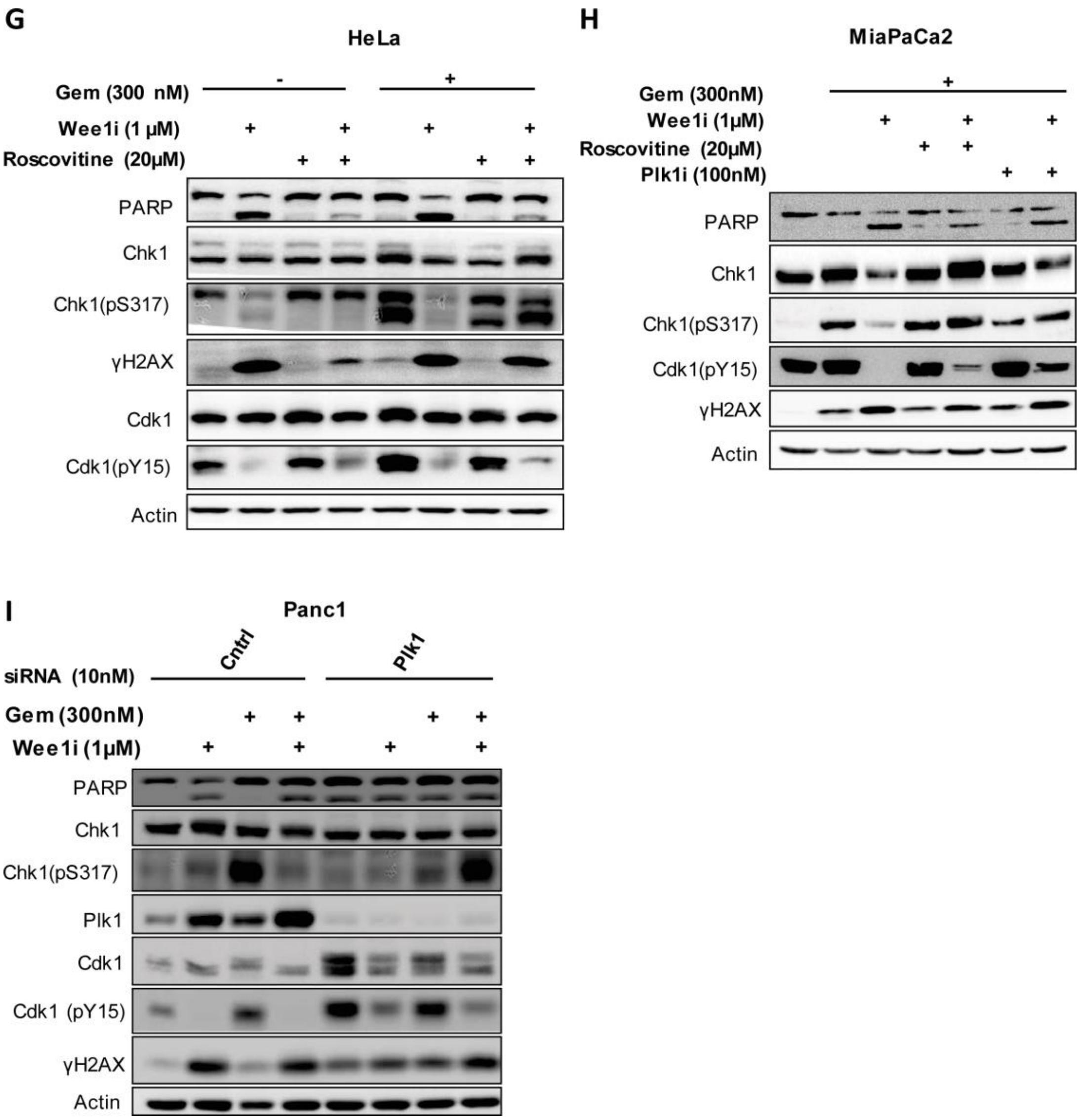

Figure S2.

(A) Depletion of Wee1 consistently decreases phosphorylation of Chk1. Another set of Wee1 siRNA (silencer) was used to remove Wee1 from the cells, confirming the results obtained in **Fig. 2C**.

(B, C) Wee1 knockdown leads to a significant decrease in phospho-Rad17 intensity. Cells of the indicated lines were transfected with siRNA against Wee1 (s21) or control siRNA (Cntrl). After 48h, the cells were treated with gemcitabine for 24h, followed by fixation, staining of phospho-Rad17 and quantitative immunofluorescence analysis as in **Fig. 3, A and B**. n=3

(D, E) Removal of Cdk1 restores the phosphorylation of Chk1 upon simultaneous knockdown of Wee1 and treatment with gemcitabine. 10nM of each siRNA (Cdk1, Wee1, scrambled) was used for transfecting cells. After 48h of transfection, gemcitabine was added for another 24h. Afterwards, cells were harvested and immunoblot analysis was performed.

(F) The levels of mRNA encoding ATR do not change significantly upon Wee1 inhibition in gemcitabine-treated cells. Panc1 cells were transfected with 10nM siRNA against Wee1. After 48 h, the cells were treated with gemcitabine for 24 h; afterwards, cells were harvested, RNA was isolated and quantitative RT-PCR was performed. 36B4 mRNA was used as a reference gene for normalization. Error bars represent standard error of the mean. The CFX manager software (Biorad) was used for the calculations.

(G) The Retinoblastoma protein is not required for the loss in Chk1 phosphorylation. HeLa cells were treated with Wee1i, gemcitabine and/or Roscovitine for 24 h. Cells were harvested and processed for immunoblot analysis as described in **Fig. 4A**.

(H) MiaPaCa2 cells show rescue of Chk1 phosphorylation upon co-inhibition of Cdk or Plk1 upon treatment with Wee1 inhibitor and gemcitabine. MiaPaCa2 cells were treated with different combinations of Wee1i, gemcitabine, roscovitine and Plk1i for 24h, followed by western blot analysis.

(I) Elimination of Plk1 in the presence of Wee1 inhibitor and gemcitabine reinstates Chk1 activity. Cells were transfected with siRNA to Plk1. 48h later, Wee1 inhibitor and/or gemcitabine were added for 24h. Cells were analyzed as to their content of phosphorylated Chk1 by immunoblotting.

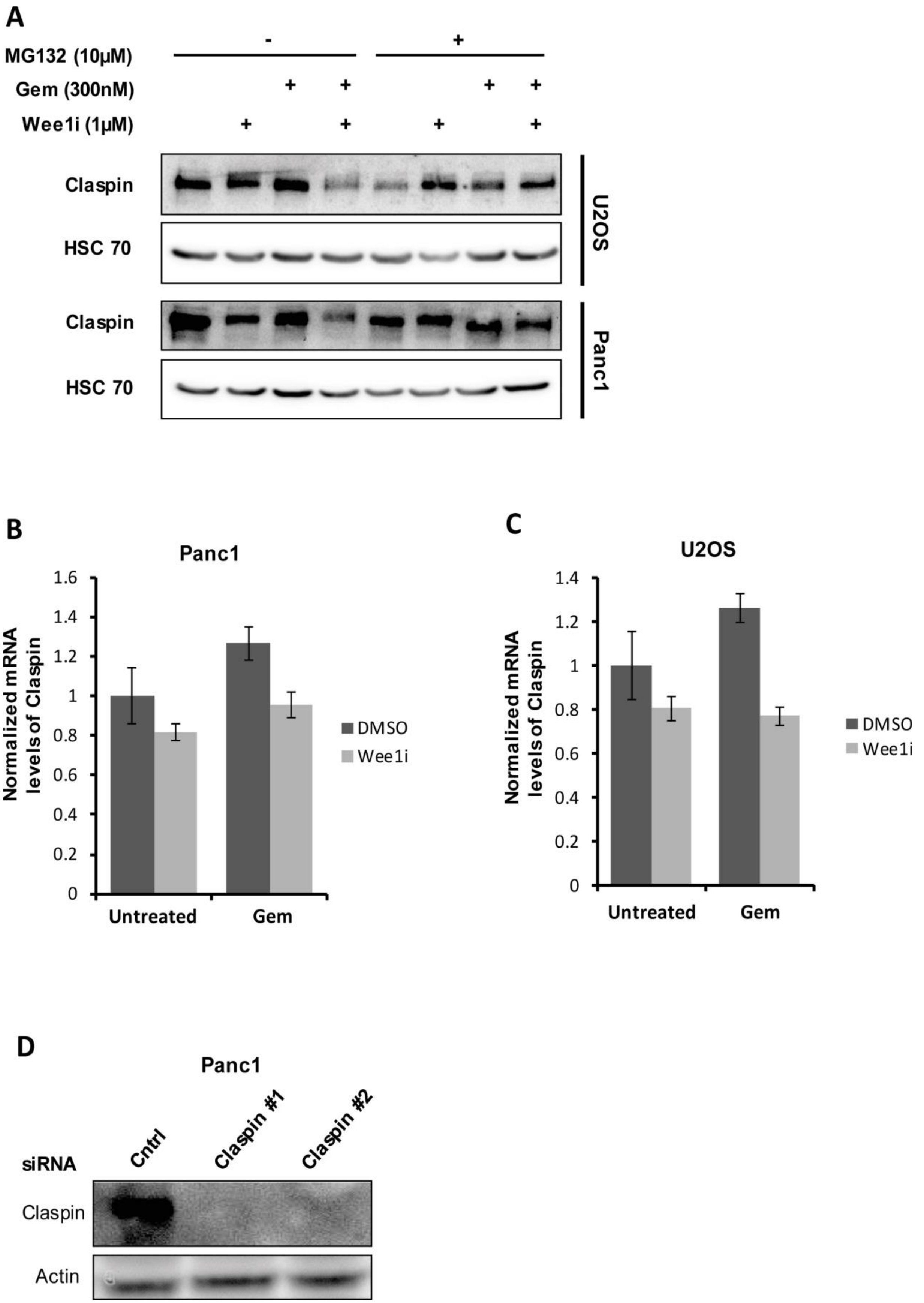
A
MG132 (10μM)
-
+
Gem (300nM)
Wee1i (1μM)
Claspin
HSC 70
U2OS
Claspin
HSC 70
Panc1
B
Panc1
Normalized mRNA levels of Claspin
1.6
1.4
1.2
1
0.8
0.6
0.4
0.2
0
Untreated
Gem
DMSO
Wee1i
C
U2OS
Normalized mRNA levels of Claspin
1.4
1.2
1
0.8
0.6
0.4
0.2
0
Untreated
Gem
DMSO
Wee1i
D
Panc1
siRNA
Cntrl
Claspin #1
Claspin #2
Claspin
Actin

Figure S3.

(A) Proteasomal degradation causes the loss of Claspin upon treatment with Wee1 inhibitor and gemcitabine. Cells of the indicated lines were treated with Wee1i and gemcitabine for 2h, followed by addition of DMSO or MG132 (a proteasome inhibitor) for another 4h. Cell lysates were immunoblotted for detecting Claspin. Of note, the claspin levels dropped in response to MG132 alone. Although difficult to explain in full, we suggest that this phenomenon might be due to a change in translation efficiency. Correspondingly, others have noticed that MAPKAPK2, a kinase induced by replicative stress [51], is capable of modulating the translation of specific mRNAs [52].

(B, C) Transcriptional regulation of Claspin does not change when Wee1 activity is blocked in the presence of gemcitabine. Cells were treated with Wee1i and gemcitabine in the presence or absence of MG132 for 4h. Afterwards, cells were harvested, RNA was isolated and quantitative RT-PCR was performed. 36B4 mRNA was used as a reference gene for normalization. Error bars represent standard error of the mean. The CFX manager software was used for the calculations.

(D) Claspin is efficiently removed by two different siRNAs. Panc1 cells were transfected with two different siRNAs against Claspin. After 48 h, cells were treated with gemcitabine for 24 h, followed by harvesting and immunoblot analysis. Negative control siRNA was used for control samples.

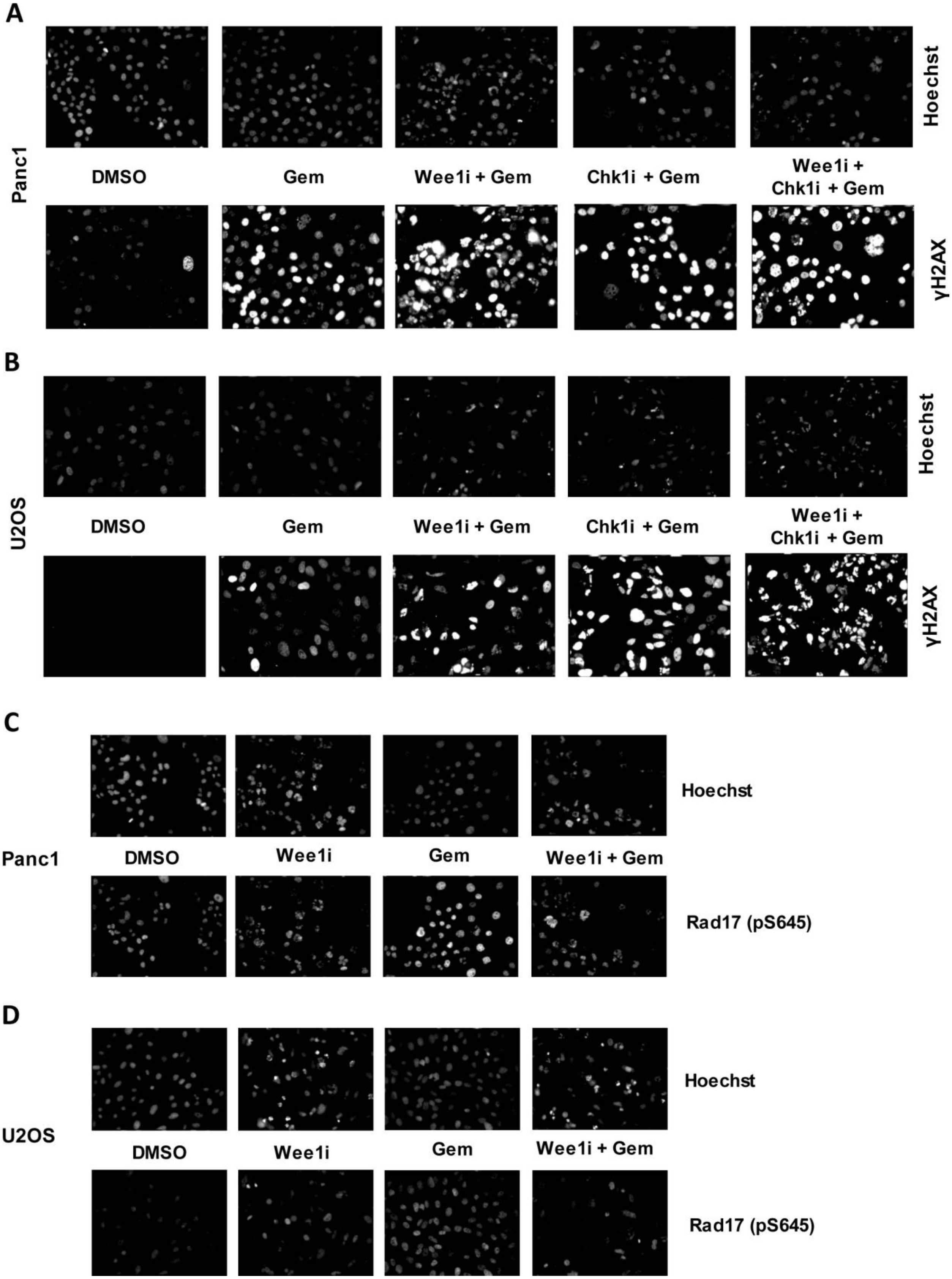
A
Panc1
DMSO
Gem
Wee1i + Gem
Chk1i + Gem
Wee1i + Chk1i + Gem
Hoechst
γH2AX
B
U2OS
DMSO
Gem
Wee1i + Gem
Chk1i + Gem
Wee1i + Chk1i + Gem
Hoechst
γH2AX
C
Panc1
DMSO
Wee1i
Gem
Wee1i + Gem
Hoechst
Rad17 (pS645)
D
U2OS
DMSO
Wee1i
Gem
Wee1i + Gem
Hoechst
Rad17 (pS645)

Figure S4.

(A, B) γH2AX intensity increases with combination of checkpoint kinase inhibitors and gemcitabine.

Panc1 and U2OS were treated for 24 h with gemcitabine, followed by treatment with checkpoint kinase inhibitors (5µM Chk1i; 1µM (Panc1) or 0.5µM (U2OS) Wee1i; 10µM ATRi) and gemcitabine for another 20 h. Cells were then fixed and stained for γH2AX. Images were taken for each treatment for quantitative analysis, and a section of each image is shown here. The quantitative assessment is shown in **Fig. 1, E and F.**

(C,D) Phospho-Rad17 intensity decreases when the Wee1 inhibitor is combined with gemcitabine. Panc1 (A) and U2OS (B) cells were treated with 1µM Wee1i or DMSO in the presence or absence of 300nM gemcitabine for 24 h. Cells were then fixed and stained for phosphorylated Rad17. Images were taken for each treatment at 20X magnification for quantitative analysis, and a section of each image is shown here. The quantitative assessment is provided in **Fig. 3, A and B.**
